# Zinc-Chelating Mechanism of Sea Cucumber (*Stichopus japonicus*)-Derived Synthetic Peptides

**DOI:** 10.3390/md17080438

**Published:** 2019-07-25

**Authors:** Xiaoyang Liu, Zixu Wang, Fawen Yin, Yuxin Liu, Ningbo Qin, Yoshimasa Nakamura, Fereidoon Shahidi, Chenxu Yu, Dayong Zhou, Beiwei Zhu

**Affiliations:** 1 School of Food Science and Technology, Dalian Polytechnic University, Dalian 116034, China; 2 National Engineering Research Center of Seafood, Dalian 116034, China; 3 Graduate School of Environmental and Life Science, Okayama University, Okayama 700-8530, Japan; 4 Department of Biochemistry, Memorial University of Newfoundland, St. John’s, NL A1B 3X9, Canada; 5 Department of Agricultural and Biosystems Engineering, Iowa State University, Ames, IA 50011, USA

**Keywords:** zinc-chelating peptide, sea cucumber, chelation mechanism

## Abstract

In this study, three synthetic zinc-chelating peptides (ZCPs) derived from sea cucumber hydrolysates with limited or none of the common metal-chelating amino-acid residues were analyzed by flame atomic absorption spectroscopy, circular dichroism spectroscopy, size exclusion chromatography, zeta-potential, Fourier transform infrared spectroscopy, Raman spectroscopy and nuclear magnetic resonance spectroscopy. The amount of zinc bound to the ZCPs reached maximum values with ZCP:zinc at 1:1, and it was not further increased by additional zinc presence. The secondary structures of ZCPs were slightly altered, whereas no formation of multimers was observed. Furthermore, zinc increased the zeta-potential value by neutralizing the negatively charged residues. Only free carboxyl in C-terminus of ZCPs was identified as the primary binding site of zinc. These results provide the theoretical foundation to understand the mechanism of zinc chelation by peptides.

## 1. Introduction

Zinc is a catalytic element that is involved in modulating a large number of enzymes, and plays important biological and structural roles in many cellular events [1]. Several factors have been found to affect zinc absorption such as inositol hexakisphosphate (phytate) that is a saturated cyclic acid in plant-based foods, which can lead to in the formation of insoluble complexes with zinc and negatively influence the intestinal zinc absorption [2,3]. To overcome the problem arisen from the interaction of zinc-phytate, an approach that biologically associates zinc with phytate competitors has been suggested as a promising way [4]. An increasing number of zinc supplements, especially bioactive peptides, with the capability to promote and enhance zinc bioavailability, are being identified and characterized. Food-derived bioactive peptides generated from proteolysis of the primary structures of proteins can exhibit diverse bioactivities, such as metal chelation and enhancement of metal absorption in the gastrointestinal system [5]. For the investigation of chelation mechanism, oligopeptides with spatially isolated binding sites have been used as promising models [6] since the affinity of chelating metal ions towards specific binding sites is largely determined by the sequence of the peptide structures [7]. Sea cucumbers contain considerable amount of trace elements, including zinc [8], in their organs and tissues, thus could be good sources for zinc-chelating peptides [9]. To fully utilize them, mechanism of chelation of zinc by sea cucumber-derived peptides needs to be characterized and understood.

Amino acid residues such as Cys [10], His [11], Asp [12] and Glu [12] in peptides are closely correlated to their metal chelating ability. Peptides containing one to four aspartyl or glutamyl residues have been found in many metal-peptide complexes [7]. In relation to zinc-chelating peptides, several particular factors, such as the carboxyl group of asparagine, quantity and distribution of hydrophilic groups, and carboxyl oxygen and amine nitrogen atoms have been suggested as being important [9,13]. The presence of carboxylates in side chains, and particular spatial location of aspartyl residues within the peptides could influence zinc chelation capability, and also simultaneously contribute to the thermodynamic stability of peptides [7]. Conventionally it is believed that metal-chelating peptides are mostly of low molecular weight (<500 Da) [13,14,15], however, recent reports reveal that some high molecular weight (>500 Da) peptides are also equally effective in their chelation ability [16,17]. Furthermore, during metal chelation process, the dimensional and geometrical alterations of secondary structures could occasionally occur in peptides [7]. Therefore, in terms of zinc-peptide chelation, the relationship between structural changes of peptides and zinc-chelating activity needs to be characterized.

An additional advantage of food-derived zinc-chelating peptides is their relatively trivial safety concerns. They are generally considered safe when used as zinc supplements for human consumption [18]. Furthermore, it is well documented that food-derived peptides could be used to characterize the mechanism of metal-peptide chelation [7,19]. However, sea cucumber derived peptides have not been systematically studied for their zinc-chelating activities. In the present study, three zinc-chelating peptides (ZCPs) were synthesized based on the amino acid sequences previously identified from hydrolysates of sea cucumber. A series of analytical methods, such as flame atomic absorption spectroscopy (FAAS), circular dichroism (CD) spectroscopy, size exclusion chromatography (SEC), zeta-potential, Fourier transform infrared (FTIR) spectroscopy, Raman spectroscopy and nuclear magnetic resonance (NMR), were used to analyze the underlying zinc chelation mechanism of these peptides. Based on these experiments, identification of zinc-binding sites of sea cucumber-derived peptides would be able to be accomplished and zinc-chelating capacity would also be improved. This work aimed to provide a mechanistic basis for analyzing the effectiveness of zinc chelating peptides from sea cucumbers, which potentially can contribute to the improvement of zinc bioavailability in human gastrointestinal system and the development of food-derived biological zinc supplements.

## 2. Results and Discussion

### 2.1. Determination of Zinc Chelating Capacity of Synthetic ZCPs

**Figure 1:**
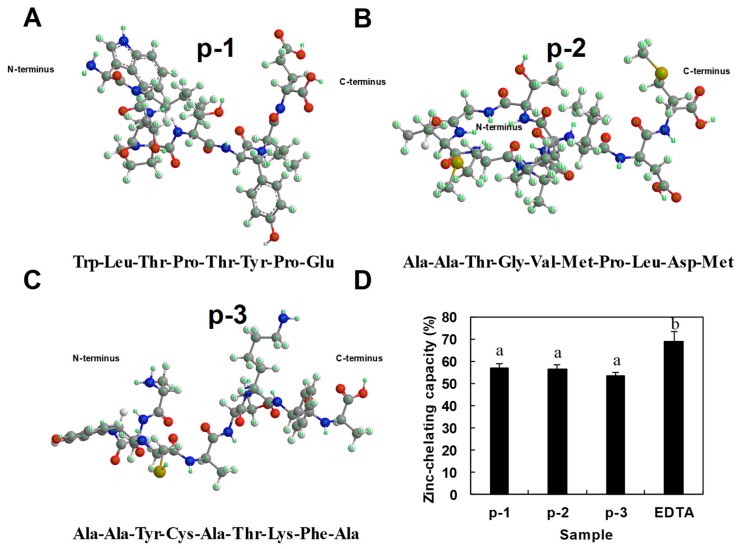
(**A**–**C**). Structures of three synthetic sea cucumber-derived peptides. (**D**). Determination of zinc-chelating capacity. Different letters above the bars indicate significant differences among fractions (I < 0.05). Values presented are the mean of triplicate analyses.

Three ZCPs (p-1, p-2 and p-3) were synthesized based on the amino acid sequences from sea cucumber from our previous work, as shown in Figure 1A–C. The amino acids at the C-terminus of the three peptides are Glu, Met and Ala, respectively. Several reports have suggested that specific amino acid residues such as Glu, His and Asp significantly contribute to zinc chelation capacity of peptides [20]. Among them, histidine residues were shown to form zinc binding motifs coordinated around their imidazole rings with high binding capacity [9,21]. However, in our synthetic peptides, other than p-1, which has a Glu on the C-terminus, the other two peptides without terminus chelating amino acid residues also exhibited zinc chelation capacity (Figure 1D). Results showed the zinc-chelating capacity of the three peptides was 56.93%, 56.80% and 53.46%, respectively. With very different amino acid compositions, the similarity in zinc binding capacity among the three ZCPs suggests that zinc chelation may be determined more by the configuration of the peptides than the influences of particular amino acids and their location. In order to better assess zinc-chelating ability of ZCPs, FAAS, a method often used for the measurement of binding between peptides and metal ions [22], was used to measure the absolute zinc-chelating capacity at three different peptide:zinc loading molar ratios. The amounts of zinc bound to the ZCPs were compared by changing the loading molar ratio of ZPCs and zinc (1:0.5, 1:1 and 1:2). The results (Table 1) showed that zinc-chelating capacity increased almost twice with the loading ratio changing from 0.5 to 1, but further increase of the loading ratio from 1 to 2 did not bring much change to the chelating capacity, indicating a possible saturation threshold for the three ZCPs for zinc chelation at a loading molar ratio of 1. Several studies have reported on the zinc-chelating capacity of naturally derived zinc-chelating peptides, such as whey protein hydrolysates (28%–25% of zinc-chelating capacity) [23] and wheat germ protein hydrolysates (86.78% of zinc-chelating capacity) [24]. Furthermore, the absolute zinc-chelating capacity (5.36 ug mg^−1^) was measured from the protein hydrolysates of oyster, which is considered to be one of the highest [9]. In comparison, the zinc-chelating capacity of our sea cucumber-derived ZCPs were reasonably good (at ~2 ug mg^−1^).

### 2.2. Evaluation of Structural Conformation of ZCPs after Chelation with Zinc

**Figure 2:**
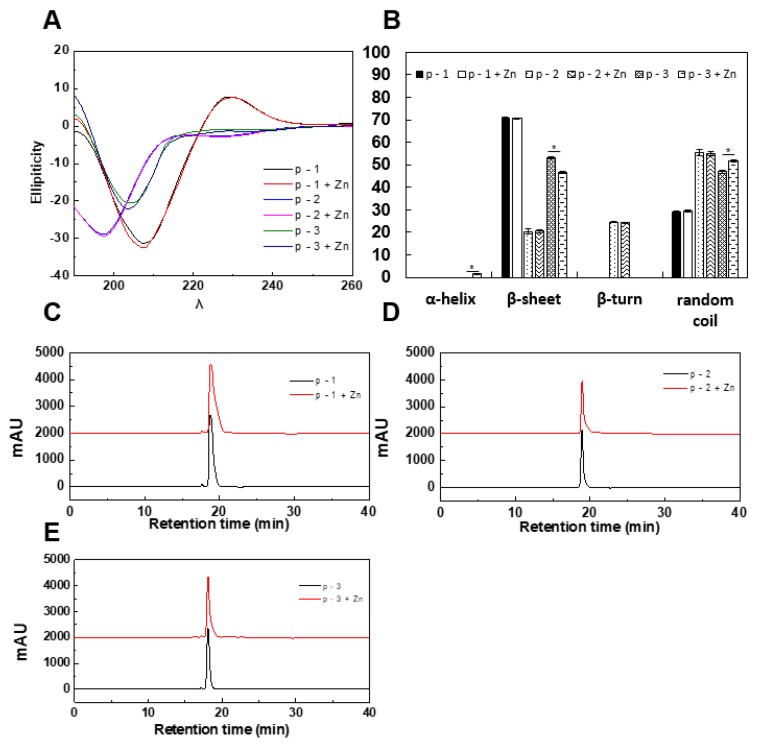
(**A**). Circular dichroism (CD) spectra of ZCPs and ZCP-zinc complexes. (**B**). Statistical analysis data of CD spectra. (**C**–**E**). Molecular weight distribution of ZCPs and ZCP-zinc complexes determined by Raman chromatography (SEC). Values presented are the mean of triplicate analyses.

The evaluation of structural changes of peptides after chelation with metals is important to understand the mechanism of chelation. The structural changes of ZCPs after chelation with zinc were analyzed by CD spectroscopy. The CD spectra (Figure 2A,B) of ZCPs showed no α-helix, but existence of β-sheets, β-turns and random coils were confirmed within the secondary structures of ZCPs. Among zinc-treated ZCPs, only p-3 showed detectable changes in β-sheets, β-turns and random coils, whereas p-1 and p-2 did not. As shown in Figure 2B, once chelated with zinc, abundances of β-sheets and random coils in p-3 changed from 53.13% ± 0.49% to 46.7% ± 0.43% and 47.1% ± 0.49% to 51.76% ± 0.55%, respectively, whereas no such change was observed for p-1 and p-2. Furthermore, zinc chelation also seemed to induce the formation of α-helix in p-3 (from 0% to 1.5% ± 0.1%). The CD results suggested that in certain ZCPs a small portion of β-sheets was converted to random coils and/or α-helixes upon binding with zinc, but it was not universal in all ZCPs. Although alteration of secondary structures of p-3 after chelation was statistically significant, the extent of changes appeared to be minimal compared with other reports [25,26]. Several reports have mentioned that, in the process of chelation between peptides and metal ions, secondary structures of peptides tend to be altered due to the formation of new bonds between metal ions and the peptides. For instance, a naturally occurring cyclic octapeptides could interact with zinc, leading to a concentration-dependent formation of zinc-peptide complex with shift of secondary structures detected by CD spectroscopy [27], including metal-induced conformation of α-helix [28]. However, some divalent or trivalent metal ions, such as Ni^2+^ or Cr^3+^, could strongly associate with the carboxyl groups in the linear peptide and prevent the amino and carboxyl groups from forming a cyclic structure [29], which means that the original secondary structures of peptides could be mostly retained after chelation with these metal ions. This might be one of the reasons why zinc binding seemed not to have a major impact on secondary structures of the three ZCPs (Figure 2A,B).

During chelation between peptides and metals, peptides could crosslink to form multimers [26]. Formation of multimers from peptides exponentially increased the molecular weight, which could be detected by SEC [30] for the range of 900–18,000 Da [31]. In this study, SEC was used to check if multimers of ZCPs were formed due to zinc chelation. Our results (Figure 2C–E) show no change in molecular weight (MW) after zinc chelation, indicating these ZCPs operated as individual units before/after zinc chelation. Furthermore, crosslinking of peptides could contribute to the shift of their secondary structures determined by CD spectroscopy [32]. Our conformational analysis using CD spectroscopy revealed that no large-scale structural change occurred for all three ZCPs upon binding with zinc, consistent with the results from SEC showing that these peptides remained monomeric (Figure 2C–E). It is worth noting that Cys residue is contained in the p-3 while disulfide bond formation would happen if peptides are in a non-reducing environment [33]. However, our results revealed that no multimers were found in p-3, which demonstrated that disulfide bond might not be formed due to possibilities of mild reaction conditions (water-mediated, 37 °C, and 30 min) and potential chemical block of Cys. In addition, ^13^C NMR could also be used to measure the formation of disulfide bond [34], which will be further investigated.

### 2.3. Evaluation of Chelation by Zeta-Potential Analysis and FTIR 

**Figure 3:**
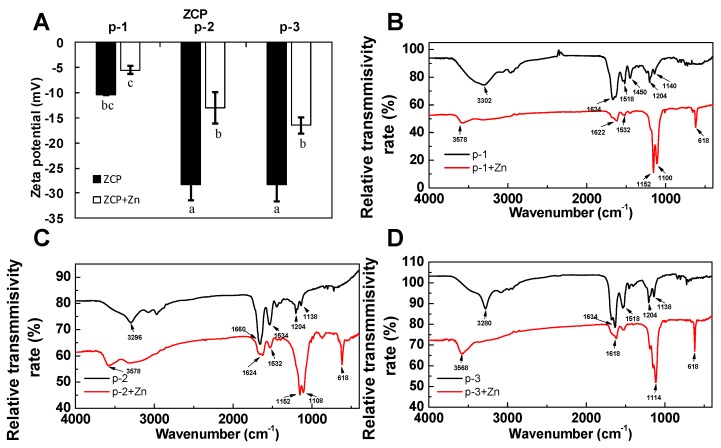
(**A**). Zeta-potential of ZCPs and ZCP-zinc complexes. (**B**–**D**). FTIR spectra of ZCPs and ZCP-zinc complexes. Different letters above the bars indicate significant differences among fractions (*p* < 0.05). Values presented are the mean of triplicate analyses.

To further investigate binding of ZCPs to zinc, and to understand the potential mechanism of electrostatic interactions between zinc and the ZCPs, zeta-potential analysis was conducted [35,36]. As shown in Figure 3A, results showed that the magnitudes of zeta-potential of p-1, p-2 and p-3, before chelating with zinc, were −10.4, −28.2 and −28.3 mv (pH = 7.0), respectively. Generally, the net repulsive electrostatic forces among particles are positively correlated to the absolute magnitudes of zeta-potential, which determine the resistance towards the formation of agglomerates [37]. The magnitudes of zeta-potential of p-2 and p-3 were higher than that of p-3, indicating their comparably higher electrostatic stability compared to p-3 in the neutral aqueous environment (they are also more negatively charged). As expected, results also showed that the zeta-potential of each of the three peptides increased to −5.48, −12.97 and −16.43 mv when zinc was added: The positively charged zinc ions neutralized some of the negative charges of the peptides (i.e., carboxyl groups). Apparently, not all of the negative charges on the ZCPs were neutralized through zinc-chelating, as shown in Figure 3A. The change in zeta potential also suggested that unwanted attenuation of stability of the ZCPs might result from zinc chelation, which reduces the magnitudes of the zeta potential to less than 30 mv [37]. Zeta-potential is sensitive to the ion strength and pH of the surrounding aqueous environment [38], further work is needed to optimize the buffer medium to ensure the stability of the ZCPs during chelation with zinc.

To identify amino acid residues or functional groups that were involved in zinc-chelation, FTIR spectra of the three peptides with or without zinc were acquired. As shown in Figure 3B–D, the conformational changes of peptides upon binding to zinc ions are reflected in band changes at 3280 to 3578 cm^−1^, 1450 to 1660 cm^−1^, 1100 to 1204 cm^−1^ and 618 cm^−1^, indicating involvement of groups, such as N–H, –COO^-^ group and C–H group, respectively. In p-1, a band at 1450 cm^−1^, which could be attributed to glutamyl carboxylate anion (Glu–COO^-^), was found to shift upon zinc binding. This band was not observed for p-2 and p-3. This suggested the –COO^−^ group of Glu in p-1 was involved in chelating zinc, which is consistent with the previous reports [39,40]. Changes in amide I band at 1634 cm^−1^ and amide II at 1532 cm^−1^ both suggest that zinc may be bound to ZCPs by interacting with carboxyl groups, similar to what has been reported for zinc-binding peptides from oyster [9]. Moreover, changes in bands associated with COOH groups, such as OH stretching vibration (3678 cm^−1^) and COO^-^ out of plane bending (618 cm^−1^) [41] indicated that the carboxyl groups were primary binding sites for zinc in these sea cucumber-derived ZCPs. Overall, based on the results of FTIR, carboxylic groups of ZCPs were primarily involved in zinc chelation.

### 2.4. Raman Spectroscopy

**Figure 4:**
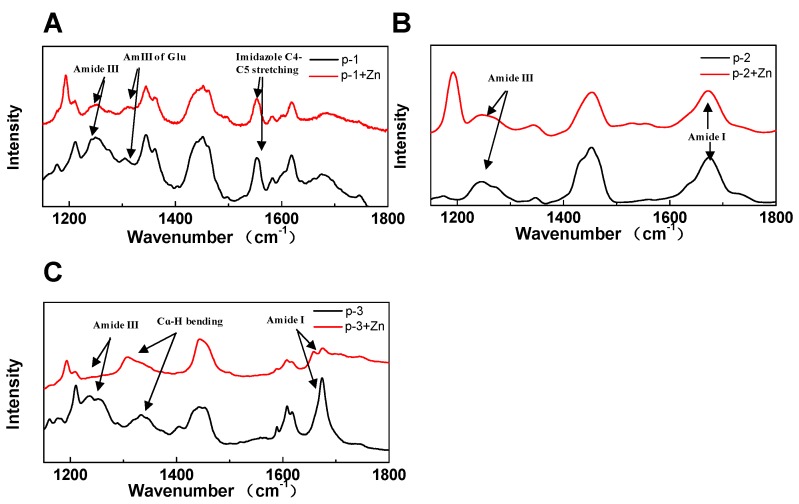
(**A**–**C**) Raman spectroscopy of ZCPs and ZCP-zinc complexes.

Raman spectroscopy was also utilized to gain insights towards the chelating mechanism of ZCPs. The Raman spectral study of three ZCPs with or without zinc were shown in Figure 4A–C. Changes in bands of amide I, Cɑ-H bending and amide III have all been associated with zinc-chelating [42,43,44]. As shown in Figure 4A–C, similar patterns were obtained for all three peptides, indicating that the primary conformational structures were not that different among them. In p-1, upon binding to zinc, amide III band (1320 cm^−1^) [45] exhibited spectral changes that could be attributed to zinc-chelating through Glu (Figure 4A). However, band of indole (1556 cm^−1^) similar to C4–C5 stretching [45] possibly arisen from Trp in p-1, showed no change after chelation with zinc, suggesting that nitrogen in indole was not the zinc-binding site (Figure 4A). This is different from previous reports about nitrogen atoms in His-containing peptides, such as Amyloid β peptide, that effectively contribute to metal ion chelation [25]. In p-2, zinc chelation did not lead to easily identifiable spectral changes (Figure 4B), consistent with the CD/SEC results (Figure 2A,B). In p-3, upon zinc-binding, changes in amide III band indicated a conformational transition of β-sheets (Figure 4C). This is also consistent with the CD data showing the decrease of β-sheets in p-3 (Figure 2A,B). Furthermore, the individual shift of Cɑ-H bending band around 1300 cm^−1^ suggested the presence of ɑ-helix like structures, also supported by the CD results (Figure 2A,B). Alongside with the FTIR data (Figure 3B–D), these results showed that ZCPs might chelate zinc primarily through carboxylate groups rather than nitrogen in indole groups. Each of the ZCPs has carboxylate group available as zinc-chelating sites, e.g., two free carboxyls in p-1 and p-2, one free carboxyl in the C-terminus of p-3, and an acylaminos group in each of them. Further investigation into exactly which carboxylate groups were involved in zinc chelating was conducted hereafter with NMR.

### 2.5. NMR 

**Figure 5:**
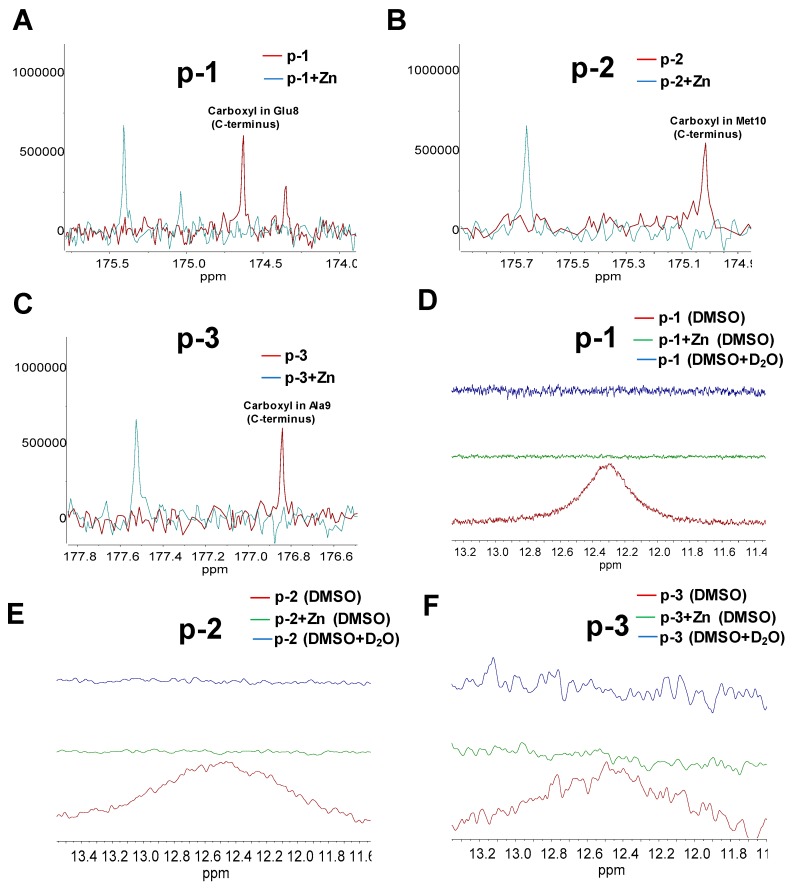
(**A**–**C**). ^13^C NMR spectra of ZCPs and ZCP-zinc complexes. (**D**–**F**). ^1^H NMR spectra of ZCPs (with or without D_2_O) and ZCP-zinc complexes.

The zinc-binding motifs in ZCPs usually are also essential for the maintenance of their secondary structures and biological properties [46]. Chemical shift deviation (CSD) in NMR can provide information about local structures in peptides [47]. Therefore, 1-D NMR was used to evaluate specific binding sites of the three ZCPs. As shown in Figure 5, ^13^C NMR spectra of p-1, p-2 and p-3 with or without zinc indicated that the primary resonance peak (170 to 180 ppm) of the three peptides could be attributed to carboxylate groups or amide carbonyl, and their correspondent quantities were discovered to be consistent with their structures, respectively (Figure S1). ^13^C NMR signals can be used to analyze the distribution of electrons around particular carbon atoms in the carboxylic groups [48]. Based on the calculated ^13^C shielding tensors, the chemical shift of carbon atoms in carboxylic group (>174 ppm) [49] is higher than that in amide carbonyl (169–174 ppm) [50,51,52]. Therefore, free carboxyls of Glu8 (176.5 and 174.5 ppm) in p-1, of Asp9 (174.8 ppm) and Met10 (175 ppm) in p-2, of Ala9 (176.9 ppm) in p-3 were identified (Figure 5A–C). In the presence of zinc, signals of carboxyls (176.5 ppm in p-1, 175 ppm in p-2 and 176.9 ppm in p-3) from each ZCP dramatically shifted, indicating that these carboxylic groups on C-terminus were involved in chelating zinc (i.e., zinc binding site in each ZCP). Furthermore, in order to confirm the involvement of carboxylic group in zinc chelation, ^1^H NMR was also conducted. As shown in Figure 5D–F, signals of carboxylic group (12.3–12.6 ppm) were identified in p-1, p-2 and p-3. The disappearance of the same signals was also discovered by addition of D_2_O, indicating the characteristic features of active hydrogen, which broadways reflected the potential zinc-binding role of carboxylic group. Results also demonstrated that zinc-chelation attenuated signals of carboxylic group (12.3–12.6 ppm) in each ZCP, indicating the involvement of carboxylic groups in zinc chelation (Figure 5D–F). However, based on the results of ^1^H NMR, it is noteworthy that the signals that represented carboxylic groups on side chain of Glu8 in p-1 and Asp9 in p-2 appeared to be involved in binding to zinc whereas the change of chemical shifts of ^13^C spectra representing these two carboxylic group in side chain was not obvious compared with those of free carboxylic group in C-terminus. Therefore, the carboxylic groups on side chain of Glu8 in p-1 and Asp9 in p-2 was not the primary zinc binding site, changes in ^1^H NMR might be because the chemical environment of hydrogen in side chains was influenced by zinc chelation of carboxylic group in C-terminus rather than chelation with zinc. Generally, for chelation with divalent metal ions, negatively charged residues, such as oxygen containing groups (carbonyl or hydroxyl) and amino groups (histidine), are the primary binding sites. Since there is no histidine in these ZCPs, and indole group was concluded not to be the zinc binding site (Figure 4A), free carboxyls at the C-terminus were found to be the primary zinc binding sites. They are essential to chelate divalent metal ions to form stable peptide-zinc complexes [51,53].

## 3. Materials and Methods

### 3.1. Materials

Zinc-chelating peptides (ZCPs, p-1, p-2 and p-3) derived from sea cucumber (*Stichopus japonicus*) hydrolysates were synthesized by ChinaPeptides Co., Ltd. (Shanghai, China). 4-(2-pyridylazo) resorcinol was purchased from Sigma-Aldrich (St. Louis, MO, USA). Dithiothreitol (DTT) was purchased from BBI Life Science (Shanghai, China). Zinc sulphate heptahydrate (ZnSO_4_.7H_2_O) was purchased from Sinopharm Chemical Reagent (Shanghai, China). Potassium bromide (KBr) was purchased from Aladdin Industrial Corporation (Shanghai, China). All other chemicals were analytical grade and used without any further purification.

### 3.2. Determination of Zinc-Chelating Capacity

The zinc-chelating capacity of the synthetic ZCPs was determined by a previously described method with some modification [23]. The principle of assay is based on the reaction that 4-(2-pyridylazo) resorcinol (PAR) can form a red complex with free zinc ions. Each of the three ZCPs was dissolved in 2-[4-(2-hydroxyethyl) piperazin-1-yl] ethane sulfonic acid (HEPES)-KOH buffer (40 mM, pH = 7.5) at 1 mg/mL with EDTA used as positive control due to its zinc-cheating ability. Each sample (250 μL, 1 mg/mL) was then mixed with 125 μL DTT (8 mM) and 125 μL ZnSO_4_ (250 μM) and incubated at 37 °C for 30 min. Then, 25 μL PAR (2 mM) were added and the absorbance was read at 500 nm. Zinc-chelating capacity (%) was calculated from the following equation:

Zinc-chelating capacity (%) = [(OD_blank_ − OD_sample_)/OD_blank_] × 100

where the blank contained all assay reagents but without any test sample of ZCP.

### 3.3. Flame Atomic Absorption Spectroscopy

Each ZCP (p-1, p-2 and p-3) was dissolved in deionized (DI) water into final concentration (1 mM), 1 mL of each was then mixed with 1 mL zinc sulfate solution with various concentration (0.5 mM, 1mM, 2mM or blank (ZCPs and DI water)) and incubated at 37 °C for 30 min. Then, to remove free zinc ions, all the samples were dialyzed in deionized water through dialysis membrane (500 Da, MYM Biological Technology Company Limited, Beijing, China) for 60 h. Volumes of all samples were adjusted to 3 mL. Zinc content of each sample was measured by flame atomic absorption spectroscopy (HITACHI, Kyoto, Japan) with an air/acetylene flame through a standard curve of zinc (data not shown). Results indicated the absolute zinc-chelating capacity of each ZCP (μg mg^−1^).

### 3.4. Circular Dichroism (CD) Spectroscopy

Each ZCP was dissolved in deionized water (1 mM), 1 mL of each was mixed with 20 mM zinc sulfate solution (1 mL) or a blank control at 37 °C for 30 min, and then measured by a circular dichroism (CD) spectrophotometer (J-1500, Jasco Corporation, Tokyo, Japan). The ratio of ZCPs and zinc was 1:20. The CD tests were carried out in high-purity nitrogen atmosphere at room temperature by using a quartz cell of 0.1 cm path length. Absorption spectra of the above samples were recorded between 190 and 260 nm using the following instrumental parameters: Bandwidth = 1.0 nm and scanning rate = 100 nm/min. Each acquired spectrum represented an average of three consecutive scans. Estimation of secondary structure was performed by using Yang’s data as reference [54].

### 3.5. Size Exclusion Chromatography (SEC)

Each ZCP was dissolved in deionized water (1 mM), and 1 mL of each was mixed with 20 mM zinc sulfate solution (1 mL) or blank control at 37 °C for 30 min. The ratio of ZCPs and zinc was 1:20. Then, 10 μL of each sample were loaded onto a TSKgel G2000SWxl (TOSOH, Kyoto, Japan) (7.8 × 300 mm). The mobile phase was acetonitrile/water/trifluoroacetic acid = 45/55/0.1 at a flow rate of 0.5 mL/min. Absorbance was monitored at 214 nm by 1260 Infinity HPLC (Agilent, Palo Alto, CA, USA).

### 3.6. Zeta-Potential Measurement 

A Nano ZS-90 (Malvern Instruments Ltd., Worcestershire, UK) was used to measure zeta potential of the ZCPs and ZCP-zinc complexes. To prepare fresh solution, each of the three ZCPs was dissolved in deionized water (1 mM), 1 mL of each was mixed with 2 mM zinc sulfate solution (1 mL) or blank control at 37 °C for 30 min. The ratio of ZCPs and zinc was 1:2. Then, all the samples were dialyzed in deionized water through dialysis membrane (500 Da) for 60 h and collected for zeta-potential measurement.

### 3.7. FTIR Spectroscopy 

Each of the three ZCPs was dissolved in deionized water (1 mM), 1 mL of each was mixed with 20 mM zinc sulfate solution (1 mL) or blank control at 37 °C for 30 min. The ratio of ZCPs and zinc was 1:20. After reaction, the samples were lyophilized. One milligram lyophilized ZCPs or ZCPs-zinc complex was mixed with 100 mg dried KBr, and the FTIR spectra were recorded using an infrared spectrophotometer (Perkin Elmer, Salem, MA, USA) from 400 to 4000 cm^−1^ at a resolution of 4 cm^−1^. The entire experimental work was conducted at room temperature.

### 3.8. Raman Spectroscopy

Each of the three ZCPs was dissolved in deionized water (1 mM), 1 mL of each was mixed with 20 mM zinc sulfate solution (1 mL) or blank control at 37 °C for 30 min. The ratio of ZCPs and zinc was 1:20. After lyophilization, samples were measured by dispersive Raman microscope (HORIBA, Kyoto, Japan) at excitation wavelength of 532 nm. A 100× objective was used. The spectra were recorded from 400 cm^−1^ to 2000 cm^−1^.

### 3.9. NMR

Each of the three ZCPs was dissolved in deionized water (5 mM), 1 mL of each was mixed with 20 mM zinc sulfate solution (1 mL) or blank control at 37 °C for 30 min. The ratio of ZCPs and zinc was 1:20. Afterwards, the samples were lyophilized and re-dissolved in d-DMSO (250 μL). Then, samples were transferred into NMR tubes. ^13^C NMR spectra of p-1, p-2 and p-3 (dissolved in D_2_O) as well as ^1^H NMR spectra (dissolved in DMSO-*d*_6_, DMSO-*d*_6_/10% D_2_O) were performed on a 400-MHz NMR spectrophotometer (Bruker, Karlsruhe, Germany).

### 3.10. Statistical Analysis

Each experiment was repeated three times. Data were presented as mean ± standard deviation (SD). One-way ANOVA was applied to analyze variance by using SPSS 16.0 software (SPSS Inc., Chicago, IL, USA) and Tukey’s test was employed to measure the differences between means. Comparisons that yielded *p* values <0.05 were considered significant.

## 4. Conclusions

**Figure 6:**
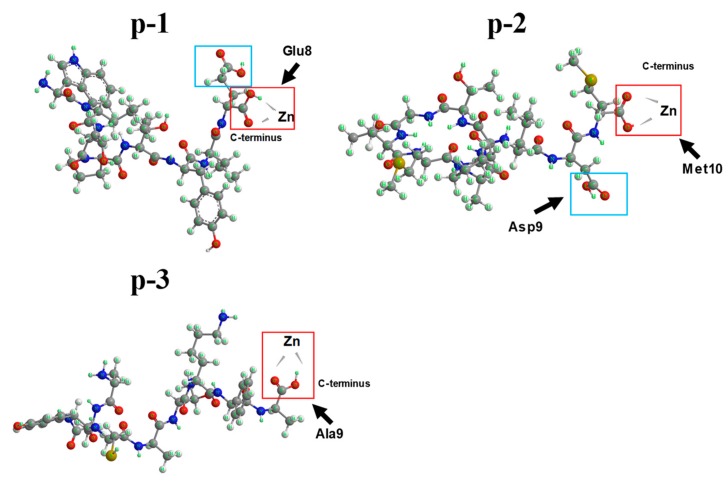
Schematic model of proposed chelation of sea cucumber-derived peptides with zinc.

Based on the results of this work, a schematic model of how these sea cucumber-derived ZCPs chelate zinc was proposed (Figure 6). In the absence of typical metal-binding residues, such as His, nitrogen-containing groups either in side chains are not involved in zinc chelation. The binding of p-1, p-2 and p-3 to zinc is primarily through interaction with free the carboxyls at C-terminus. As a conclusion, it is shown that zinc-chelating peptides do not necessarily have to have classical metal-chelating amino acid residues. Zinc chelation can rely on binding with free carboxyl in the C-terminus of peptides, which suggests a much larger scope of peptides could be effective zinc-chelating agents. Overall, based on the results, this study might offer theoretical bases to the development of approaches that contribute to obtain peptides with rich zinc-binding site and improve the zinc-chelating capacity, which could contribute to industrial manufacture of zinc supplements in the future. Further investigation is thus needed to explore how these peptides help to improve zinc bioavailability.

## Figures and Tables

**Table 1:** The absolute zinc-chelating capacity of zinc-chelating peptides (ZCPs).

ZCP:Zinc (c:c)	p-1 (ug mg^−1^)	p-2 (ug mg^−1^)	p-3 (ug mg^−1^)
1:0.5	1.10 ± 0.01a	0.88 ± 0.01a	1.54 ± 0.19a
1:1	2.34 ± 0.03a	2.03 ± 0.07a	2.01 ± 0.27ab
1:2	2.27 ± 0.05b	1.91 ± 0.11b	2.03 ± 0.03b

Absolute zinc-chelating capacity of ZCPs in different loading molar ratio of peptides and zinc. Different letters within a column above the bars indicate significant differences among each ZCP (*p* < 0.05). Means ± SD (*n* = 3).

## Supplementary Materials

The following are available online at https://www.mdpi.com/1660-3397/17/8/438/s1, Figure S1: ^13^C NMR spectra of ZCPs and ZCP-zinc complexes from 160 to 180 ppm. Numbers represent designated carboxyl and acylamino, Figure S2: Full spectra of ^13^C NMR spectra of ZCPs and ZCP-zinc complexes, Figure 3: ^1^H NMR spectra of ZCPs and ZCP-zinc complexes (dissolved in DMSO-d6 or DMSO-d6/10%D2O). Numbers represent designated hydrogen.

## Abbreviations

ZCP	zinc-chelating peptide
DI water	Distilled water
CSD	Chemical shift deviation
PAR	4-(2-pyridylazo)
FAAS	Flame Atomic Absorption Spectroscopy
CD	Circular Dichroism
SEC	Size Exclusion Chromatography
FTIR	Fourier Transform infrared spectroscopy
NMR	Nuclear Magnetic Resonance
MW	molecular weight
