# Supplementary material for: Zinc-Chelating Mechanism of Sea Cucumber (Stichopus japonicus)-Derived Synthetic Peptides
Source: Mar Drugs. 2019 Jul 25;17(8):438. doi: 10.3390/md17080438 (PMC6723998; doi:10.3390/md17080438)
Supplement: Supplementary file 1 [file marinedrugs-17-00438-s001.pdf]

Supporting information for:

Zinc-chelating mechanism of sea cucumber (*Stichopus japonicus*)-derived synthetic peptides

Xiaoyang Liu[1,2], Zixu Wang[1], Fawen Yin[1,2], YuXin Liu[1,2], Ningbo Qin[1,2], Yoshimasa Nakamura[3], Fereidoon Shahidi[4], Chenxu Yu[2,5], Dayong Zhou[1,2,*], Beiwei Zhu[1,2]

[1] School of Food Science and Technology, Dalian Polytechnic University, Dalian, China, 116034

[2] National Engineering Research Center of Seafood, Dalian, PR China, 116034

[3] Graduate School of Environmental and Life Science, Okayama University, Okayama, Japan, 700-8530.

[4] Department of Biochemistry, Memorial University of Newfoundland, St. John's, NL A1B 3X9, Canada.

[5] Department of Agricultural and Biosystems Engineering, Iowa State University, IA 50011, USA

Corresponding Authors:

Dayong Zhou[1,2,*]. E-mail: zdyzf1@163.com; Tel: +86-411 86323453

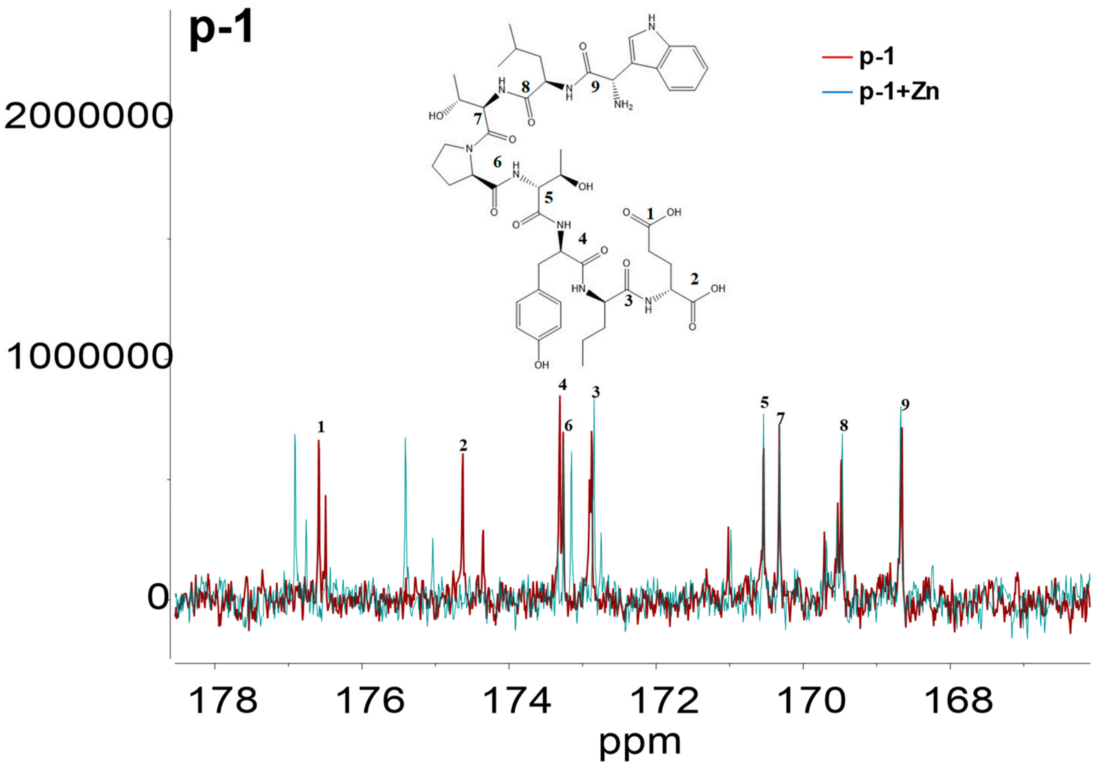
p-1
p-1
p-1+Zn
2000000
1000000
0
178
176
174
172
170
168
ppm

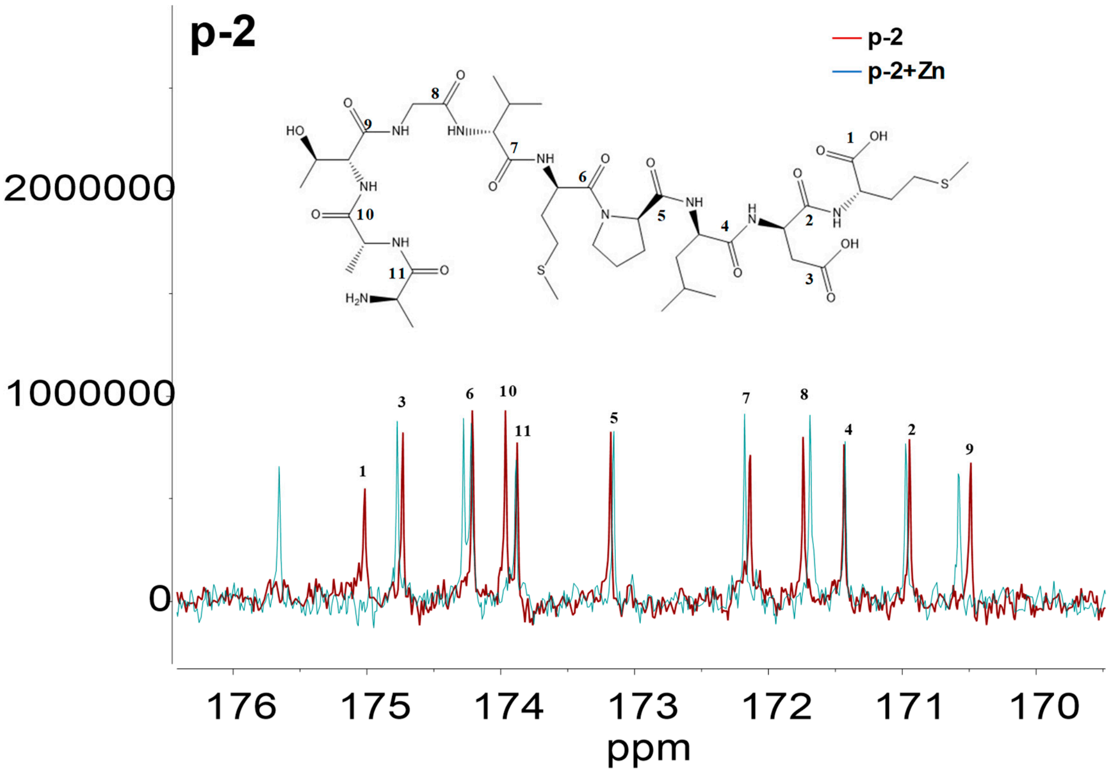
p-2
p-2
p-2+Zn
2000000
1000000
0
176
175
174
173
172
171
170
ppm

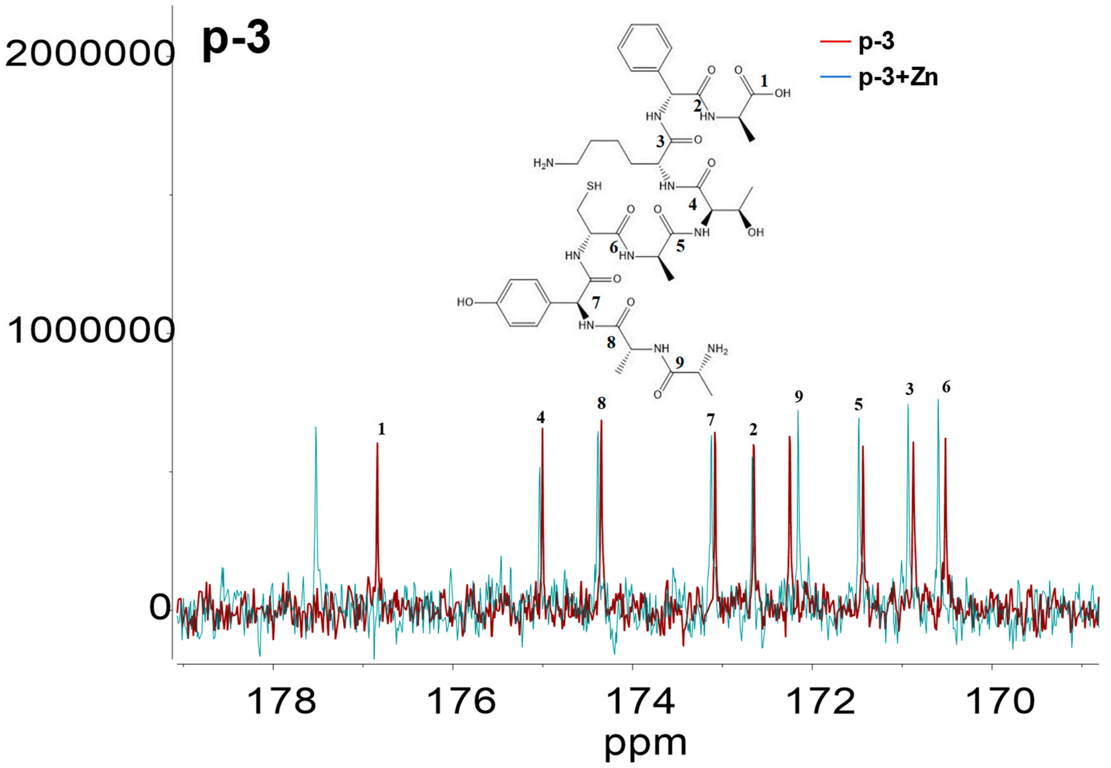

Figure. S1

^{13}C NMR spectra of ZCPs and ZCP-zinc complexes from 160 to 180 ppm. Numbers represent designated carboxyl and acylamino.

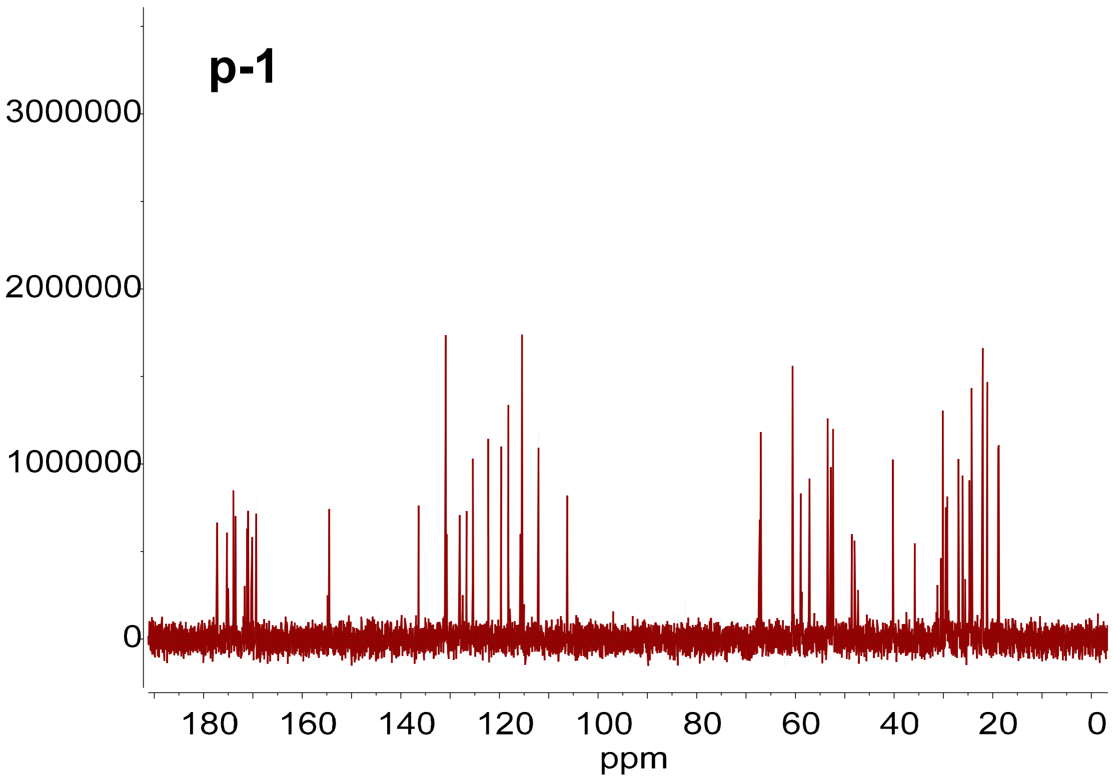

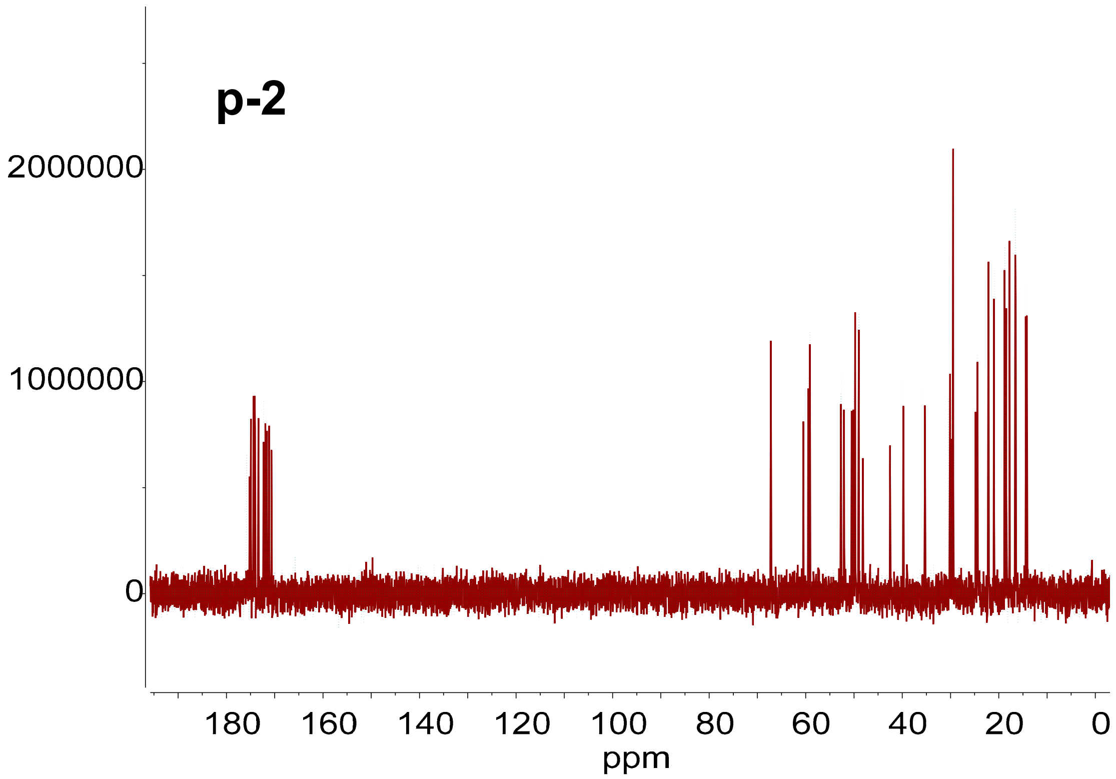

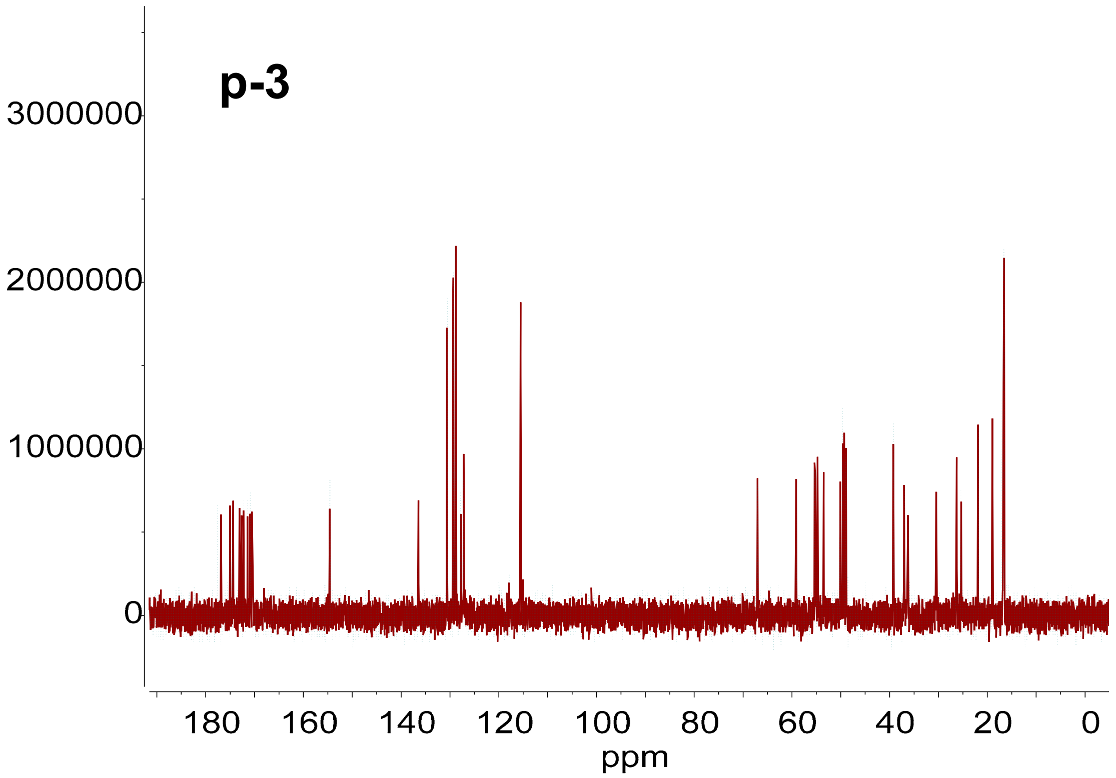

Figure.S2

Full spectra of ^{13}C NMR spectra of ZCPs and ZCP-zinc complexes.

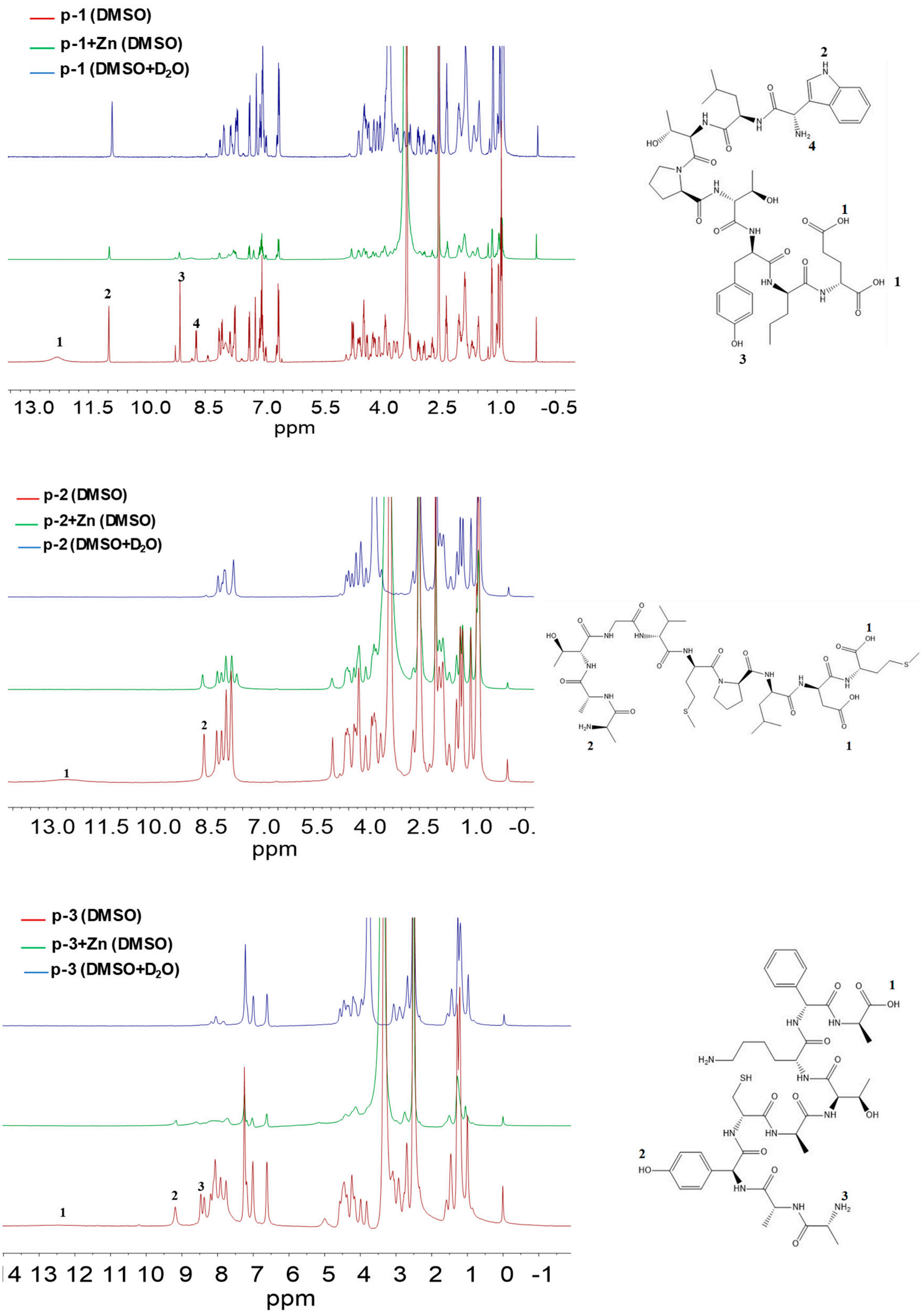

Figure.S3

^{1}H NMR spectra of ZCPs and ZCP-zinc complexes (dissolved in DMSO-d6 or DMSO-d6/10%D_2O). Numbers represent designated hydrogen.
